# Effect of Anticholinergic Drug Burden on Postoperative Delirium in Elderly Patients: A Nested Case–Control Study

**DOI:** 10.1002/cns.70731

**Published:** 2026-01-04

**Authors:** Ting Zhang, Tianqi Shen, Ningxin Li, Xiaoying Zhang, Kai Zhang, Chang Liu, Bingbing Meng, Shaohua Zhang, Guangyu Tang, Ziyi Zhang, Qiang Fu, Yanhong Liu, Jingsheng Lou, Jiangbei Cao, Weidong Mi, Hao Li

**Affiliations:** ^1^ Medical School of Chinese People's Liberation Army General Hospital (PLA) Beijing China; ^2^ Department of Anesthesiology, the First Medical Center, Chinese PLA General Hospital Beijing China; ^3^ National Clinical Research Center for Geriatric Diseases Chinese PLA General Hospital Beijing China; ^4^ Department of Anesthesiology and Combat Trauma Center No. 984 Hospital of the PLA Beijing China

**Keywords:** aged, anticholinergic drug, case–control study, delirium, postoperative cognitive complications

## Abstract

**Background:**

Postoperative delirium (POD) is a common complication in elderly patients. This study aimed to investigate the association between preoperative anticholinergic drug burden and POD in elderly patients.

**Methods:**

This nested case–control study included patients aged ≥ 65 years who underwent general anesthesia between April 2020 and April 2022 at multiple hospitals in China. POD occurring within 7 days postoperatively was assessed using the 3‐Minute Diagnostic Interview for Confusion Assessment Method. Preoperative anticholinergic drug burden was quantified using the Anticholinergic Cognitive Burden (ACB) scale. Univariate and multivariate logistic regression models with random effects were used to determine the association between ACB scores and POD occurrence. Kaplan–Meier survival analysis with log‐rank tests was plotted to compare the cumulative POD incidence across groups. Subgroup analyses were performed to explore the relationship between ACB scores and POD occurrence within specific populations.

**Results:**

Among 10,296 patients, 1131 (11.0%) developed POD. The study employed a 5:1 matched case–control design and included 1125 cases and 5296 matched controls. Univariate (odds ratio [OR]: 1.230; 95% confidence interval [CI]: 1.119–1.353, *p* < 0.001) and multivariate (adjusted OR: 1.118; 95% CI: 1.006–1.243, *p* = 0.037) analyses demonstrated a significant association between higher anticholinergic drug burden and increased POD risk. When analyzed categorically (ACB score 0 as reference), adjusted ORs were 1.100 (95% CI: 0.919–1.317; *p* = 0.296) for ACB = 1, 1.213 (95% CI: 0.831–1.771; *p* = 0.318) for ACB = 2, and 1.963 (95% CI: 1.253–3.076; *p* = 0.003) for ACB ≥ 3. Kaplan–Meier analysis demonstrated a significantly higher cumulative incidence of POD in the ACB ≥ 3 group (log‐rank *p* < 0.001), with divergence starting on postoperative day 3.

**Conclusion:**

A higher preoperative anticholinergic drug burden is associated with an increased risk of POD in elderly patients, particularly when the ACB scores are ≥ 3.

## Graphical abstract

This nested cohort study, nested within a multicentre prospective dataset of 6421 elective non‐cardiac surgery patients aged ≥ 65 years, tested whether pre‐operative anticholinergic burden predicts incident post‐operative delirium. Multivariable conditional logistic regression analysis revealed that each 1‐point increment in the Anticholinergic Cognitive Burden (ACB) score independently raised delirium odds by 10%; patients accumulating an ACB score ≥ 3 experienced a 77% higher delirium risk.

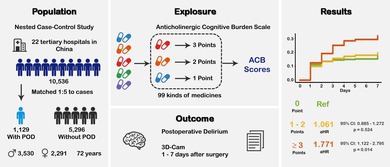

## 1 Introduction

The aging global population presents a growing challenge to public health systems, with an increasing number of elderly patients requiring surgical treatment. Perioperative complications in the elderly are common and often associated with poor prognoses, making their prevention a major clinical priority [1]. Postoperative delirium (POD) is an acute, transient neurocognitive disorder that typically arises within the first week after surgery. It is characterized by inattention, fluctuating consciousness, and abrupt cognitive dysfunction in elderly patients [2, 3]. A large‐scale multicenter study involving > 20,000 elderly surgical patients across 30 hospitals reported a POD incidence of 12.0%, with rates rising to 13.7%–57.3% after cardiac surgery [4, 5]. Similarly, a multicenter prospective study of elderly patients undergoing noncardiac surgery reported an incidence of 12.8% [6]. Although usually reversible, POD is associated with serious long‐term consequences, including persistent cognitive decline [7, 8, 9], prolonged hospitalization [10], increased hospital readmission, and higher mortality rates [11]. Thus, identifying modifiable risk factors and recognizing high‐risk patients are critical to enhancing postoperative recovery and long‐term outcomes in older surgical patients.

Elderly individuals often use medications with anticholinergic properties to manage conditions such as allergies, asthma, hypertension, depression, Parkinson's disease, and cardiovascular disorders [12], leading to a cumulative anticholinergic burden [13]. Side effects include visual disturbances, dry mouth, heat intolerance, constipation, urinary retention, cognitive impairment, and tachycardia [14]. While typically mild in younger individuals, these effects are often more pronounced in older adults due to age‐related changes in drug metabolism and clearance, as well as a natural decline in central cholinergic neurons [15, 16]. Several studies have reported an association between anticholinergic exposure in elderly patients and elevated risk of POD [17, 18] and cognitive decline [19, 20, 21]. However, evidence remains inconsistent. Some studies suggest that a high preoperative anticholinergic burden increases POD risk in elderly patients [17, 22], while others report no significant association, particularly in those without pre‐existing cognitive impairment [23, 24]. Considering these contradictory findings, further well‐designed studies are warranted.

This prospective multicenter study aimed to evaluate the association between preoperative anticholinergic drug burden and the incidence of POD in elderly surgical patients. The findings aim to provide critical evidence to guide clinical decision‐making regarding anticholinergic medication management during the perioperative period.

## 2 Patients and Methods

### 2.1 Study Design

This nested case–control study was derived from the Perioperative Database of Chinese Elderly Patients Study, a prospective multicenter observational cohort. This study was approved by the Institutional Review Board of the First Medical Center of the Chinese PLA General Hospital (Approval No. S2024‐854‐01) and registered at ClinicalTrials.gov (registration no. NCT06931353). We examined geriatric patients (aged ≥ 65 years) who developed POD following major surgery. This study adhered to the Strengthening the Reporting of Observational Studies in Epidemiology guidelines [25] and the Declaration of Helsinki following the Committee on Publication Ethics guidelines to ensure methodological transparency.

### 2.2 Data Sources

The data were drawn from the Perioperative Database of Chinese Elderly Patients study (ClinicalTrials.gov identifier: Registration No. NCT04911530; Ethics Approval No.: S2019‐311‐03 from the Ethics Committee of Chinese PLA General Hospital). The parent study prospectively enrolled patients aged ≥ 65 years undergoing elective noncardiac surgery at multiple tertiary centers in China between April 2020 and April 2022, with written informed consent obtained from all participants.

For the current nested case–control analysis, we excluded patients meeting any of the following criteria: (1) American Society of Anesthesiologists (ASA) physical status classification of V; (2) emergency surgery; (3) procedures lasting ≤ 30 min; or (4) datasets with > 5% missing variables.

### 2.3 Exposures

The primary exposure was anticholinergic drug burden, quantified using the Anticholinergic Cognitive Burden (ACB) scale (types of drugs are shown in Table S1) [26]. Preoperative medications were collected through structured interviews by trained researchers and included both prescription and over‐the‐counter drugs. Long‐term medication use was defined as daily use for chronic conditions (e.g., diabetes mellitus or hypertension). The ACB scale classifies 99 medications into four levels of anticholinergic potency: 0 for no anticholinergic activity to 3 for strong anticholinergic activity. The cumulative ACB score of each patient was calculated as the sum of the ACB scores of all long‐term medications. For analysis, ACB scores were treated both as continuous variables (to examine dose–response relationships) and as categorical variables (to identify potential clinical cutoffs). This dual approach enabled evaluation of both linear trends and threshold effects between anticholinergic burden and POD occurrence.

### 2.4 Covariates

Covariates were categorized into five domains: (1) Demographic characteristics (age, sex, BMI, ASA physical status [I–IV], smoking status, alcohol consumption, and education level); (2) preoperative comorbidities (hypertension, coronary heart disease, and chronic pain syndrome); (3) preoperative medications: benzodiazepines, nonsteroidal anti‐inflammatory drugs [NSAIDs], and statins; (4) laboratory parameters (hemoglobin, serum albumin, alanine aminotransferase (ALT), aspartate aminotransferase (AST), creatinine, blood urea nitrogen, and electrolytes [potassium, sodium, and calcium]); and (5) intraoperative variables (surgical duration, blood loss, urine output, volume of crystalloids administered, and perioperative use of benzodiazepines and NSAIDs).

### 2.5 Outcome

The primary outcome was POD, assessed using the validated 3‐Minute Diagnostic Interview for Confusion Assessment Method (3D‐CAM) [27]. Trained research nurses conducted daily assessments twice daily (once between 8:00 and 10:00 AM and again between 6:00 and 8:00 PM) for 7 days or until hospital discharge (whichever occurred earlier). POD was defined as at least one positive 3D‐CAM result during these assessments. The specific postoperative day of delirium onset was also recorded.

### 2.6 Generation of the Matched Cohort

All eligible patients who developed POD were designated as cases. For each case, up to five controls without POD were selected using a stratified matching approach. Controls were matched exactly by sex, within a one‐year age caliper, and within a 1 kg/m^2^ range for body mass index (BMI) [28].

### 2.7 Statistical Analyses

Data were managed using WPS Office 20,305 and analyzed using R software (version 4.4.2). Continuous variables were assessed for normality using the Kolmogorov–Smirnov test. Normally distributed data were expressed as mean ± standard deviation and compared using independent *t*‐tests; non‐normally distributed data were expressed as median (interquartile range [IQR]) and compared using Mann–Whitney *U* tests. Categorical variables were expressed as counts (percentages) and compared using χ^2^ or Fisher's exact tests, as appropriate.

Missing data were imputed using median or mean values for descriptive analyses, and multiple imputations (five iterations) were used for multivariate models. Associations were reported as odds ratios (OR), adjusted odds ratios (aOR), or hazard ratios (aHRs) with 95% confidence intervals (CI) from pooled estimates.

Univariate conditional logistic regression was initially used to evaluate the association between ACB scores and POD. Candidate variables were selected on the basis of both statistically significant between‐group differences (*p* < 0.05) and clinical relevance to POD. These variables were then entered in a stepwise manner within three predefined blocks (patient demographics, pre‐operative characteristics, and intra‐operative variables) into a multivariate conditional logistic regression model to control potential confounding. Receiver operating characteristic (ROC) curves were constructed for the final model, and the area under the ROC curve (AUROC) was used to evaluate model discrimination.

Subgroup analyses were performed to examine the potential interactions between ACB scores and key variables, such as age, education level, and baseline cognitive function.

Finally, based on the optimal ACB score cutoff, patients were categorized into risk groups. Kaplan–Meier survival curves with log‐rank tests were used to compare cumulative POD incidence across groups. Conditional Cox proportional hazards models were used to adjust for all previously identified significant covariates, with results expressed as adjusted hazard ratios (aHR) and 95% CI. Statistical significance was set at a two‐tailed *p* < 0.05.

## 3 Results

### 3.1 Patient Characteristics

**FIGURE 1:**
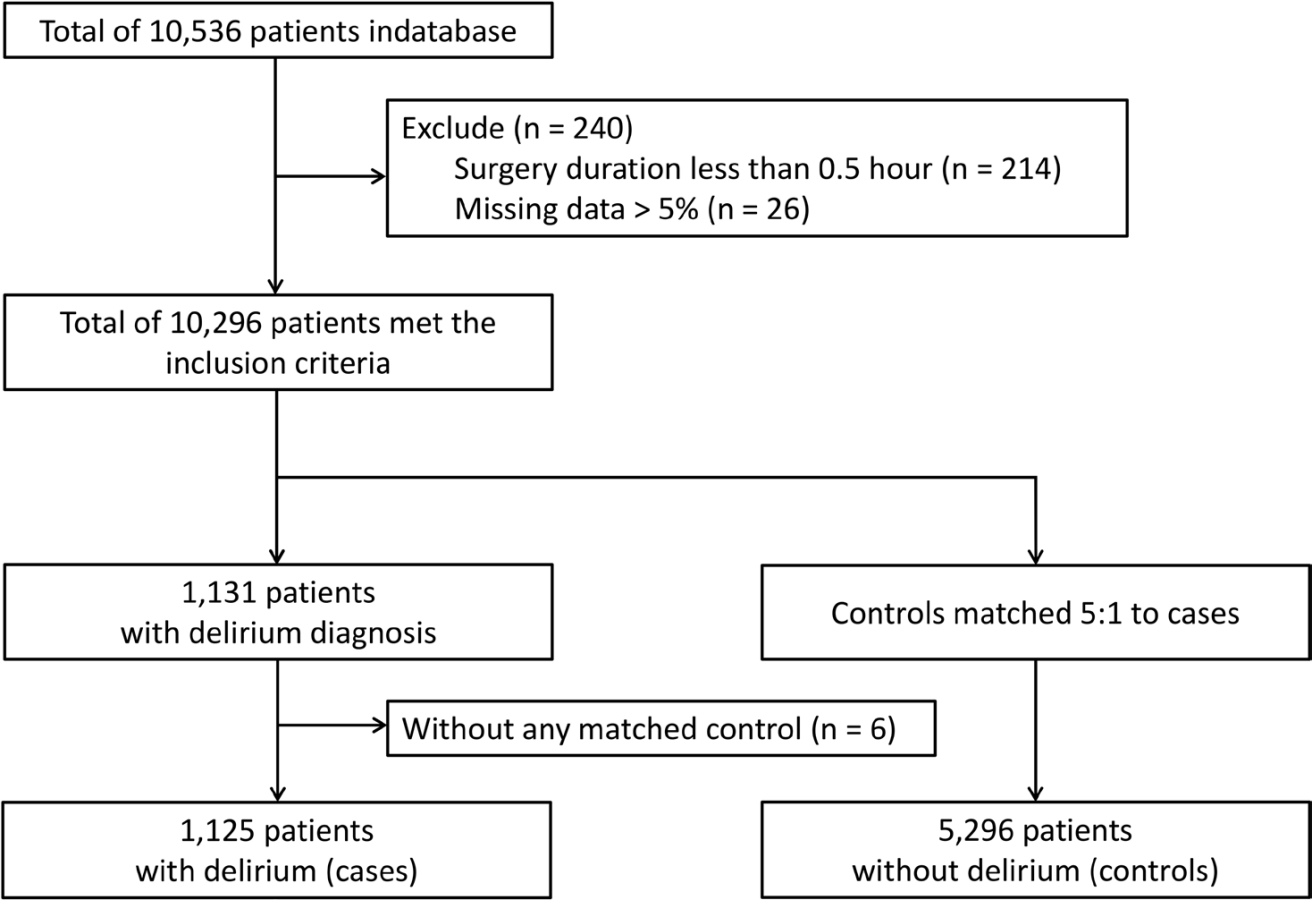
Flowchart of the patient selection process.

A total of 10,296 patients were initially enrolled. Figure 1 outlines the patient selection process. POD occurred in 1131 patients (11.0%), with six excluded due to matching failure. The final analytical cohort included 1125 POD cases and 5296 matched controls.

Table 1 summarizes the baseline characteristics of the delirium and non‐delirium groups. In the matched cohort, the control group included 2925 males (55.2%), with a median age of 72.0 years (IQR, 68.0–76.0) and a median BMI of 24.0 kg/m^2^ (IQR, 21.7–26.0). Compared with the full cohort, the matched cohort exhibited a more balanced distribution of key characteristics.

**TABLE 1:** Baseline characteristics of postoperative delirium cases and matched controls.

Variables *n* (%); M (Q_1_, Q_3_)	Full sample (*n* = 10,296)			Matched sample (*n* = 6421)		
	Control (*n* = 9165)	Case (*n* = 1131)	SMD	Control (*n* = 5296)	Case (1125)	SMD
Sex			0.064			0.029
Male	5192 (56.7)	605 (53.5)		2925 (55.2)	605 (53.8)	
Female	3973 (43.3)	526 (46.5)		2371 (44.8)	520 (46.2)	
Age, years	71.0 (68.0, 75.0)	72.0 (69.0, 77.0)	0.305	72.0 (68.0, 76.0)	72.0 (69.0, 77.0)	0.125
Height, cm	164.0 (158.0, 170.0)	163.0 (157.0, 170.0)	0.084	164.0 (158.0, 170.0)	163.0 (157.0, 170.0)	0.061
Weight, kg	65.0 (58.0, 72.0)	63.0 (56.0, 70.6)	0.095	64.0 (57.0, 71.7)	63.0 (56.0, 70.0)	0.052
BMI, kg/m^2^	24.1 (22.07, 26.4)	24.0 (21.6, 26.0)	0.053	24.0 (21.7, 26.0)	24.0 (21.6, 26.0)	0.016
ASA physical status classification			0.126			0.106
I	168 (1.8)	9 (0.8)		91 (1.7)	9 (0.8)	
II	6474 (70.7)	766 (67.7)		3659 (69.1)	763 (67.8)	
III	2431 (26.5)	347 (30.7)		1480 (28.0)	344 (30.6)	
IV	92 (1.0)	9 (0.8)		66 (1.2)	9 (0.8)	
Education level			0.129			0.136
Illiteracy or unknown	1786 (19.5)	265 (23.4)		1007 (19.0)	263 (23.4)	
High school or below	5632 (61.4)	674 (59.6)		3254 (61.4)	670 (59.5)	
College	1694 (18.5)	191 (16.9)		1006 (19.0)	191 (17.0)	
Postgraduate or above	53 (0.6)	1 (0.1)		29 (0.6)	1 (0.1)	
Current smoker	2335 (25.5)	278 (24.6)	0.021	1262 (23.8)	278 (24.7)	0.021
Current drinker	2104 (23.0)	242 (21.4)	0.038	1139 (21.5)	242 (21.5)	< 0.001
Hypertension	4488 (49.0)	596 (52.7)	0.075	2610 (49.3)	591 (52.5)	0.065
Coronary heart disease	1646 (18.0)	222 (19.6)	0.043	984 (18.6)	220 (19.6)	0.025
Chronic pain	919 (10.0)	106 (9.4)	0.022	526 (9.9)	105 (9.3)	0.020
Preoperative benzodiazepines	1387 (15.1)	224 (19.8)	0.123	768 (14.5)	223 (19.8)	0.141
Preoperative NSAIDs	602 (6.6)	82 (7.3)	0.027	339 (6.4)	82 (7.3)	0.035
Preoperative statin	603 (6.6)	61 (5.4)	0.050	359 (6.8)	61 (5.4)	0.057
Hemoglobin, g/L	130.0 (119.0, 141.0)	125.0 (113.0, 138.0)	0.247	130.0 (118.0, 140.0)	126.0 (113.0, 138.0)	0.202
White blood cell, ×10^9^	5.8 (4.8, 7.1)	5.9 (4.8, 7.1)	0.039	5.8 (4.8, 7.1)	5.9 (4.9, 7.1)	0.037
Blood glucose, mmol/L	5.3 (4.8, 6.1)	5.3 (4.8, 6.1)	0.027	5.3 (4.8, 6.1)	5.3 (4.8, 6.1)	0.018
Triglyceride, mmol/L	1.2 (0.9, 1.7)	1.2 (0.9, 1.7)	0.017	1.2 (0.9, 1.7)	1.2 (0.9, 1.6)	0.020
Albumin, g/L	40.0 (37.4, 42.7)	38.9 (35.9, 41.8)	0.272	39.9 (37.2, 42.6)	38.9 (35.9, 41.8)	0.237
Blood urea nitrogen, mmol/L	5.5 (4.6, 6.7)	5.5 (4.5, 6.8)	0.003	5.5 (4.6, 6.8)	5.5 (4.5, 6.8)	0.002
Creatinine, μmol/L	71.0 (60.3, 83.6)	71.1 (60.5, 84.4)	0.064	71.0 (60.0, 83.8)	71.1 (60.4, 84.4)	0.063
Serum potassium, mmol/L	4.0 (3.8, 4.2)	4.0 (3.7, 4.2)	0.014	3.99 (3.76, 4.24)	4.0 (3.7, 4.2)	0.002
Serum sodium, mmol/L	141.4 (139.9, 142.9)	141.0 (139.4, 142.7)	0.167	141.4 (139.8, 142.9)	141.00 (139.40, 142.60)	0.157
Serum calcium, mmol/L	2.3 (2.2, 2.3)	2.3 (2.2, 2.3)	0.085	2.3 (2.2, 2.3)	2.3 (2.2, 2.3)	0.075
Total bilirubin, μmol/L	10.2 (7.5, 14.0)	10.4 (7.3, 14.5)	0.162	10.2 (7.5, 13.9)	10.4 (7.3, 14.5)	0.164
Alanine aminotransferase, U/L	15.1 (11.0, 22.0)	14.8 (10.5, 22.0)	0.077	15.0 (10.9, 21.7)	14.8 (10.6, 22.0)	0.086
Aspartate aminotransferase, U/L	18.0 (14.7, 23.0)	18.1 (14.5, 24.2)	0.122	18.0 (14.6, 23.0)	18.1 (14.5, 24.2)	0.122
Type of anesthesia			0.049			0.056
General anesthesia	1073 (11.7)	115 (10.2)		620 (11.7)	112 (10.0)	
Others	8092 (88.3)	1016 (89.8)		4676 (88.3)	1013 (90.0)	
Urine output, mL	300.0 (100.0, 600.0)	400.0 (200.0, 700.0)	0.205	300.0 (100.0, 600.0)	400.0 (200.0, 700.0)	0.219
Intraoperative blood loss, mL	50.0 (20.0, 200.0)	100.0 (50.0, 200.0)	0.209	50.0 (20.0, 200.0)	100.0 (50.0, 200.0)	0.215
Crystalloid administration, mL	1108.0 (1000.0, 1700.0)	1600.0 (1100.0, 2100.0)	0.349	1100.0 (1000.0, 1608.0)	1600.0 (1100.0, 2100.0)	0.371
Surgery duration, hours	2.3 (1.5, 3.3)	2.9 (1.8, 4.2)	0.377	2.2 (1.5, 3.3)	2.9 (1.8, 4.2)	0.397
Perioperative NSAIDs	3221 (35.2)	406 (35.9)	0.016	1831 (34.6)	406 (36.1)	0.032
Perioperative benzodiazepines	4653 (50.8)	574 (50.8)	< 0.001	2634 (49.7)	571 (50.8)	0.020

Abbreviations: ASA, American Society of Anesthesiologists; BMI, body mass index; NSAIDs, nonsteroidal anti‐inflammatory drugs.

### 3.2 Association With POD

**FIGURE 2:**
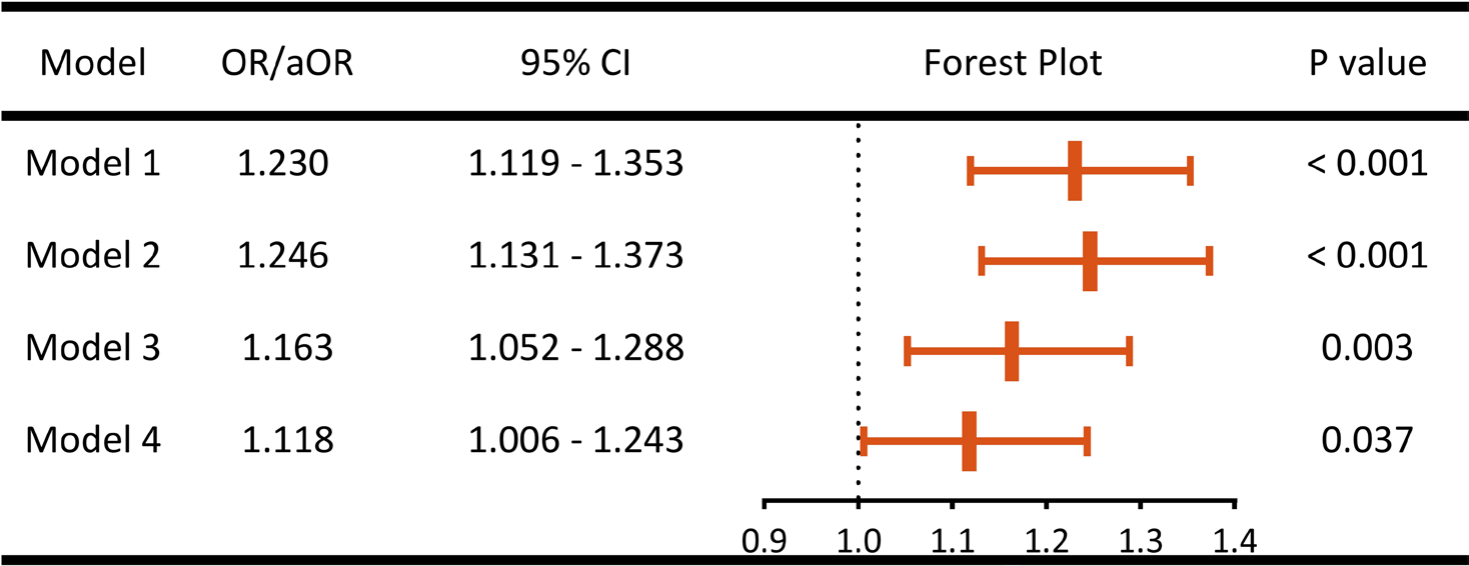
Univariate and multivariate conditional logistic regression analyses. Model 1: Univariate analysis; Model 2: Model 1 + patient‐related factors (age, sex, BMI, education level, ASA classification); Model 3: Model 2 + preoperative factors (hemoglobin, albumin, preoperative benzodiazepine, sodium, and calcium); Model 4: Model 3 + intraoperative factors (surgical duration, intraoperative benzodiazepine, blood loss, and urine output). aOR, adjusted odds ratios; ASA, American Society of Anesthesiologists; BMI, body mass index; CI, confidence intervals; OR, odds ratios.

Univariate conditional logistic regression analysis revealed a significant association between higher ACB scores and increased risk of POD (OR: 1.230, 95% CI: 1.119–1.353, *p* < 0.001). This association remained significant after sequential adjustment for potential confounders. In the fully adjusted model, we controlled for age, education, ASA grade, BMI, hemoglobin, albumin, serum sodium, serum calcium, preoperative benzodiazepine use, operative duration, intra‐operative blood loss, intraoperative urine output, and intraoperative benzodiazepine administration. Each 1‐point increase in ACB score was independently associated with a 10% higher risk of POD (aOR: 1.118, 95% CI: 1.006–1.243, *p* = 0.037), indicating a robust dose–response relationship across all models (Figure 2). Detailed specifications of the final model are presented in Table S2. The ROC curve showed that the final model exhibited good discriminative ability, with an AUROC of 0.682 (95% CI: 0.666–0.699). Moreover, the deviance residuals plot revealed no potential outliers, underscoring the robustness of the model (Figure S1).

**FIGURE 3:**
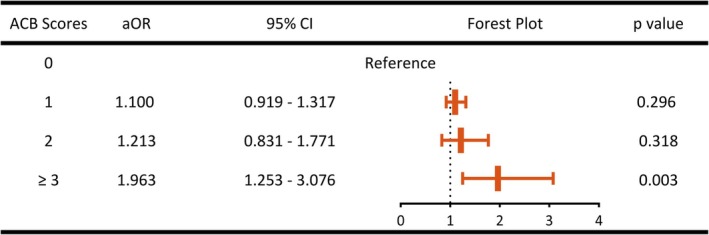
Multivariable conditional logistic regression analysis of POD with ACB scores as categorical variables. ACB, anticholinergic cognitive burden; aOR, adjusted odds ratio; CI, confidence interval.

When ACB scores were analyzed as categorical variables (0, 1, 2, and ≥ 3; ACB score 0 as reference), no significant associations were observed for ACB = 1 (aOR: 1.110, 95% CI: 0.919–1.317, *p* = 0.296) or ACB = 2 (aOR: 1.213, 95% CI: 0.831–1.771, *p* = 0.318). However, patients with ACB ≥ 3 had a significantly increased risk of POD (aOR: 1.963; 95% CI: 1.253–3.076, *p* = 0.003), suggesting a threshold effect (Figure 3).

**FIGURE 4:**
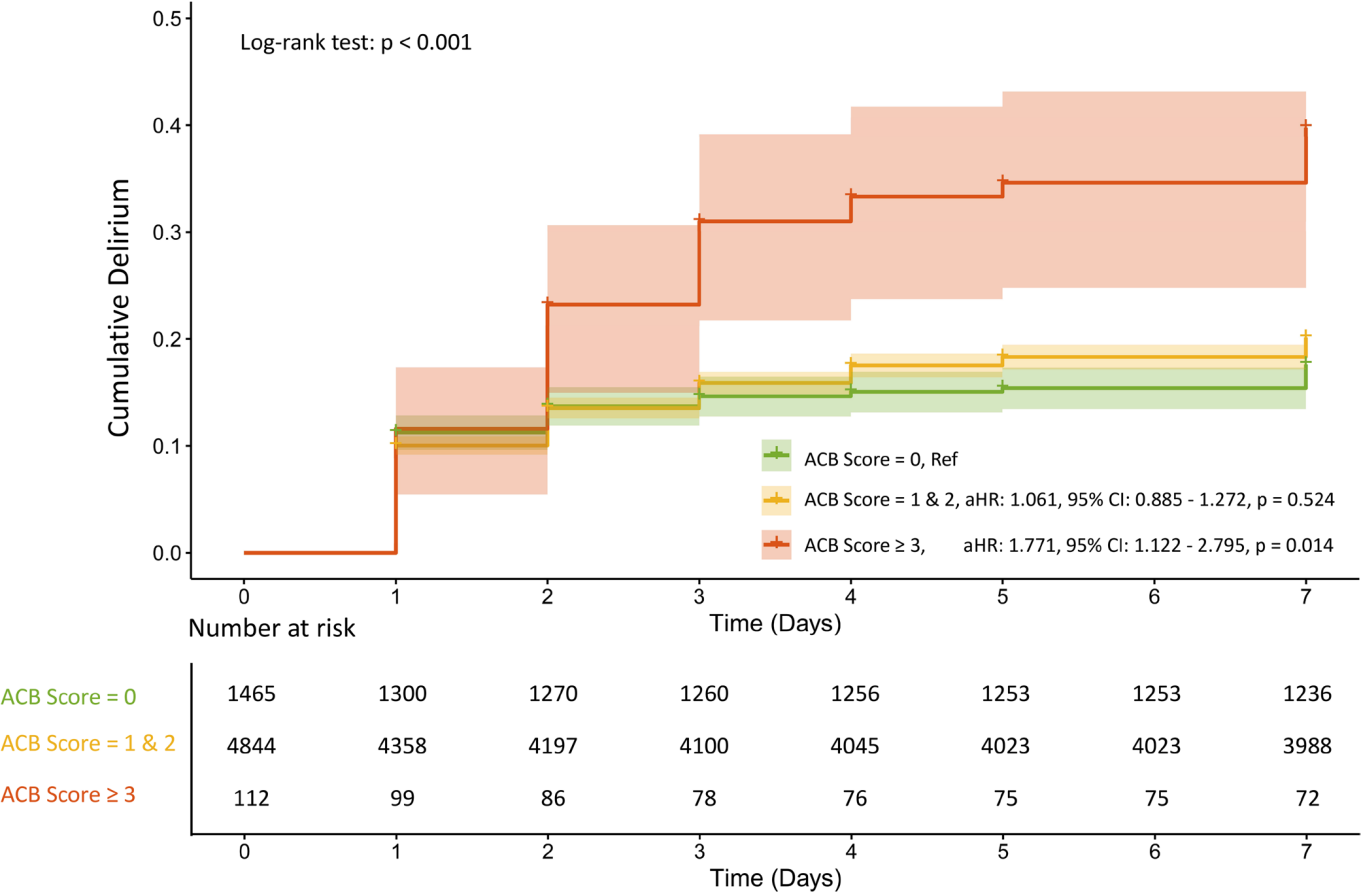
Kaplan–Meier curves for cumulative incidence of postoperative delirium by ACB scores. ACB, anticholinergic cognitive burden; aHR, adjusted hazard ratios; CI, confidence intervals.

In the multivariable conditional Cox regression analysis, ACB scores 1–2 were not significantly associated with POD risk compared to ACB = 0 (aHR: 1.061, 95% CI: 0.885–1.272, *p* = 0.524). However, ACB ≥ 3 was associated with a 77% increased hazard of delirium (aHR: 1.771, 95% CI: 1.122–2.795, *p* = 0.014). Kaplan–Meier analysis demonstrated a significantly higher cumulative incidence of POD in the ACB ≥ 3 group (log‐rank *p* < 0.001), with divergence beginning at postoperative Day 3 and persisting thereafter (Figure 4).

### 3.3 Subgroup Analyses

Subgroup analyses stratified continuous variables (age, preoperative hemoglobin, albumin, and surgical duration) using clinically relevant diagnostic thresholds or median values, and examined categorical variables (education level and preoperative benzodiazepine use). While the strength of association between ACB scores and POD varied across subgroups, no statistically significant interaction effects were observed (all interaction *p* > 0.05). The consistent effect sizes across these subgroups support the robustness of the ACB‐POD association, indicating minimal modification by patient or perioperative factors (Figure S2).

## 4 Discussion

In this prospective multicenter cohort study involving 10,536 patients, the overall incidence of POD was 10.8%. Multivariate analysis, adjusted for potential confounders, identified a significant association between preoperative anticholinergic drug burden and POD. This association became more pronounced at an ACB score of ≥ 3, highlighting the need for caution when prescribing anticholinergic medications to elderly patients.

Anticholinergic burden has been linked to long‐term cognitive consequences, including dementia, cognitive decline, and delirium [29, 30, 31]. Prior studies have identified anticholinergic burden as an independent risk factor for POD and its severity [32]. Mueller et al. [17] reported that long‐term use of anticholinergic drugs increased POD risk in elderly patients, using the Anticholinergic Drug Scale (ADS) for assessment. A prospective randomized cohort study, involving 899 elderly patients aged over 70 years, demonstrated that higher preoperative anticholinergic drug exposure measured by Anticholinergic Risk Scale (ARS) or Anticholinergic Burden Scale (ABS) was independently associated with POD [33]. Another retrospective cross‐sectional study that utilized the ACB scale revealed a positive correlation between anticholinergic drug burden and POD; however, the study population consisted solely of end‐of‐life patients [34]. Our findings are consistent with those of previous studies but offer broader applicability. Unlike prior work limited to specific surgical populations, our research focused on elderly patients undergoing elective noncardiac surgery under general anesthesia, encompassing a broader range of surgical procedures and enhancing the generalizability of the results.

Multiple tools mentioned above are available to assess preoperative anticholinergic burden. The ADS was developed from observational data on serum anticholinergic activity in elderly patients from long‐term care facilities [35], while the ARS is based on expert consensus and validated in a cohort of United States veterans aged ≥ 65 years [36]. Furthermore, the ACB scale used in our research, which includes 99 medications with established anticholinergic activity, has demonstrated strong external validity and widespread clinical utility based on its established criteria (receptor binding/serum anticholinergic activity) and comprehensive evaluation of anticholinergic effects [37, 38, 39, 40]. Research findings indicate that end‐of‐life patients with a preoperative ACB score ≥ 4 have a 2–3 times higher risk of developing delirium compared to those with an ACB score of 0 [34]. End‐of‐life patients receiving palliative care, whether in hospice settings or at home, were often prescribed multiple medications, resulting in a significantly higher anticholinergic burden. Another retrospective study involving 385 patients > 65 years undergoing orthopedic and trauma surgery also confirmed that a high preoperative anticholinergic drug burden (ACB ≥ 3) was significantly correlated with POD compared to no burden [22]. Consisting with this study, our results confirmed a significant association between higher anticholinergic burden (ACB ≥ 3) and increased risk of POD in elderly patients, which being applicable to a wider range of surgical procedures.

These findings may be attributed to the pharmacologic mechanism of anticholinergic drugs, which block acetylcholine neurotransmission by binding to muscarinic receptors [41, 42]. Acetylcholine is essential for cognitive functions, such as learning and memory. Dysregulation of the cholinergic system, including neuronal loss, acetylcholine receptor dysfunction, and impaired signaling, can lead to cognitive impairment proportional to the extent of cholinergic disruption [43, 44]. Moreover, elevated anticholinergic burden can disrupt immune homeostasis and blood–brain barrier integrity, promoting inflammation and allowing neurotoxic substances to enter the brain, thereby increasing the risk of POD [41, 45]. Despite these harmful effects, anticholinergic drugs remain widely prescribed, primarily because of their efficacy and limited awareness among physicians regarding safer alternatives. Our findings emphasize the importance of caution when prescribing anticholinergic drugs to elderly patients. When an elderly patient's anticholinergic burden score is ≥ 3, substituting alternative medications to reduce their anticholinergic burden may help lower the risk of delirium.

Although this study utilized a relatively large multicenter prospective database, it has several limitations. First, the ACB score could not be further stratified to identify a more definitive inflection point with stronger statistical significance. Second, although we adjusted for multiple covariates, unmeasured confounders may have influenced the results. Third, despite standardized and systematic training, inter‐institutional variability in POD assessment with missing cases of mild delirium may have introduced bias. Future randomized controlled trials are needed to establish the critical preoperative ACB threshold that increases POD risk.

In conclusion, our study demonstrated that a higher preoperative anticholinergic drug burden is associated with increased risk of POD occurrence in elderly patients, particularly at ACB score ≥ 3. Further randomized controlled trials are required to establish a definitive dose–response relationship between preoperative anticholinergic burden and POD, and inform medication adjustments aimed at reducing the incidence of POD.

## Author Contributions

**Ting Zhang:** conceptualization, methodology, software, validation, project administration, formal analysis, data curation, writing – original draft, writing – review and editing. **Tianqi Shen, Ningxin Li:** conceptualization, methodology, software, validation, formal analysis, data curation, writing – original draft, writing – review and editing. **Xiaoying Zhang, Kai Zhang, Chang Liu:** software, formal analysis, visualization. **Bingbing Meng, Shaohua Zhang, Guangyu Tang, Ziyi Zhang:** formal analysis, data curation. **Qiang Fu, Yanhong Liu, Jingsheng Lou, Jiangbei Cao, Weidong Mi:** methodology, validation, formal analysis, writing – review and editing. **Hao Li:** conceptualization, validation, methodology, software, writing – review and editing. All authors have provided final approval for the version to be published and collectively assume responsibility for the integrity of the work as a whole, committing to the prompt and thorough investigation and resolution of any issues that may arise concerning its accuracy or completeness.

## Disclosure

The authors have nothing to report.

## Ethics Statement

This study was approved by the Institutional Review Board of the First Medical Center of the Chinese PLA General Hospital (Approval No. S2024‐854‐01) and registered at ClinicalTrials.gov (registration no. NCT06931353).

## Conflicts of Interest

The authors declare no conflicts of interest.

## Supporting information


**Figure S1:** ROC curve and deviance residuals plot of final model. AUROC, area under the receiver operating characteristic; ROC, receiver operating characteristic.
**Figure S2:** Results of subgroup analysis. ALB, albumin; aOR, adjusted odds ratios; CI, confidence intervals; HGB, hemoglobin; OR, odds ratios.
**Table S1:** Anticholinergic cognitive burden (ACB) scale.
**Table S2:** Detailed specifications of the final model.

## Data Availability

The data that support the findings of this study are available from the corresponding author upon reasonable request.
