# Supplementary material for: Effect of Anticholinergic Drug Burden on Postoperative Delirium in Elderly Patients: A Nested Case–Control Study
Source: CNS Neurosci Ther. 2026 Jan 4;32(1):e70731. doi: 10.1002/cns.70731 (PMC12765989; doi:10.1002/cns.70731)
Supplement: Supplementary file 1 — Figure S1: ROC curve and deviance residuals plot of final model. AUROC, area under the receiver operating characteristic; ROC, receiver operating characteristic. Figure S2: Results of subgroup analysis. ALB, albumin; aOR, adjusted odds ratios; CI, confidence intervals; HGB, hemoglobin; OR, odds ratios. Table S1: Anticholinergic cognitive burden (ACB) scale. Table S2: Detailed specifications of the final model. [file CNS-32-e70731-s001.zip › cns70731-sup-0001-FigureS1-S2@Figure Legends for Supplementary Figures.docx]

**Figure Legends for Supplementary Figures**

Figure s1. ROC curve and deviance residuals plot of final model.

Abbreviations: ROC, Receiver Operating Characteristic; AUROC, Area Under the Receiver Operating Characteristic

Figure s2. Results of subgroup analysis.

Abbreviations: OR,odds ratios; aOR, adjusted odds ratios; CI, confidence intervals; HGB, Hemoglobin; ALB, Albumin
