# Supplementary material for: Effect of Anticholinergic Drug Burden on Postoperative Delirium in Elderly Patients: A Nested Case–Control Study
Source: CNS Neurosci Ther. 2026 Jan 4;32(1):e70731. doi: 10.1002/cns.70731 (PMC12765989; doi:10.1002/cns.70731)
Supplement: Supplementary file 1 — Figure S1: ROC curve and deviance residuals plot of final model. AUROC, area under the receiver operating characteristic; ROC, receiver operating characteristic. Figure S2: Results of subgroup analysis. ALB, albumin; aOR, adjusted odds ratios; CI, confidence intervals; HGB, hemoglobin; OR, odds ratios. Table S1: Anticholinergic cognitive burden (ACB) scale. Table S2: Detailed specifications of the final model. [file CNS-32-e70731-s001.zip › cns70731-sup-0003-TableS1.docx]

Table S1 Anticholinergic Cognitive Burden (ACB) scale

| Score 1 | alimemazine、alprazolam、alverine、aripiprazole、asenapine、atenolol、brompheniramine maleate、bupropion、captopril、cetirizine、chlorthalidone、cimetidine、clidinium、chlordiazepoxide、codeine、colchicine、warfarin、desloratadine、diazepam、digoxin、dipyridamole、disopyramide、fentanyl、fluvoxamine、furosemide、haloperidol、hydralazine、hydrocortisone、iloperidone、isosorbide、loperamide、loratadine、metoprolol、morphine、nifedipine、paliperidone、prednisone、quinidine、ranitidine、risperidone、theophylline、trazodone、triamterene、venlafaxine |
| --- | --- |
| Score 2 | amantadine、alkaloids、carbamazepine、cyclobenzaprine、cyprohetadine、loxapine、pethidine、methotrimeprazine、molindine、nefopam、oxcarbazepine、pimozide |
| Score 3 | amitriptyline、amoxapine、atropine、benztropine、brompheniramine、carbinoxamine、chlorpheniramine、chlorpromazine、clemastine、clomipramine、clozapine、darfenacin、desipramine、dicyclomine、dimenhydrinate、diphenhydramine、doxepin、fesoterodine、flavoxate、imipramine、meclizine、nortriptyline、olanzaoine、orphenadrine、oxybutynin、paroxetine、perphenazine、procyclidine、promazine、prometheline、propantheline、pyrilamine、quetiapine、hydroxyzine、scopolamine、solifenacin、thioridazine、tolterodine、trifluoperazine |
