# Supplementary material for: Effect of Anticholinergic Drug Burden on Postoperative Delirium in Elderly Patients: A Nested Case–Control Study
Source: CNS Neurosci Ther. 2026 Jan 4;32(1):e70731. doi: 10.1002/cns.70731 (PMC12765989; doi:10.1002/cns.70731)
Supplement: Supplementary file 1 — Figure S1: ROC curve and deviance residuals plot of final model. AUROC, area under the receiver operating characteristic; ROC, receiver operating characteristic. Figure S2: Results of subgroup analysis. ALB, albumin; aOR, adjusted odds ratios; CI, confidence intervals; HGB, hemoglobin; OR, odds ratios. Table S1: Anticholinergic cognitive burden (ACB) scale. Table S2: Detailed specifications of the final model. [file CNS-32-e70731-s001.zip › cns70731-sup-0004-TableS2.docx]

| Covariate | Coefficients | Standard Error | aOR | aOR (95% CI) | p value | VIF |
| --- | --- | --- | --- | --- | --- | --- |
| ACB Scores | 0.112 | 1.119 | 1.119 | 1.007-1.243 | 0.037 | 1.074 |
| Age | 0.948 | 2.581 | 2.581 | 1.916-3.476 | <0.001 | 1.058 |
| Education level |  |  |  |  |  | 1.005 |
| Illiteracy or unknown |  |  | Reference | | |  |
| High school or below | -0.181 | 0.835 | 0.835 | 0.705-0.986 | 0.035 |  |
| College | -0.264 | 0.768 | 0.768 | 0.615-0.958 | 0.019 |  |
| Postgraduate or above | -1.926 | 0.146 | 0.146 | 0.019-1.126 | 0.065 |  |
| ASA physical status classification |  |  |  |  |  | 1.013 |
| Ⅰ |  |  | Reference | | |  |
| Ⅱ | 0.485 | 1.624 | 1.625 | 0.807-3.268 | 0.174 |  |
| Ⅲ | 0.427 | 1.533 | 1.533 | 0.757-3.104 | 0.236 |  |
| Ⅳ | -0.247 | 0.781 | 0.781 | 0.284-2.145 | 0.632 |  |
| BMI | -0.567 | 0.137 | 0.568 | 0.434-0.742 | <0.001 | 1.030 |
| Hemoglobin | -0.004 | 0.996 | 0.996 | 0.992-1.001 | 0.085 | 1.137 |
| Albumin | -0.028 | 0.972 | 0.972 | 0.955-0.990 | 0.002 | 1.129 |
| Serum calcium | -0.175 | 0.840 | 0.840 | 0.569-1.239 | 0.379 | 1.011 |
| Serum sodium | -0.024 | 0.976 | 0.976 | 0.954-0.998 | 0.035 | 1.021 |
| Preoperative benzodiazepines | 0.338 | 1.402 | 1.402 | 1.169-1.682 | <0.001 | 1.038 |
| Surgery duration | 0.218 | 1.243 | 1.244 | 1.185-1.305 | <0.001 | 1.229 |
| Intraoperative blood loss(per 100mL) | 0.015 | 1.015 | 1.015 | 0.994-1.037 | 0.170 | 1.107 |
| Urine output(per 100mL) | 0.008 | 1.008 | 1.008 | 0.990-1.026 | 0.377 | 1.147 |
| Perioperative benzodiazepines | 0.005 | 1.005 | 1.005 | 0.874-1.156 | 0.943 | 1.051 |
| Table S2. Detailed specifications of the final model.  Abbreviations: VIF, variance inflation factor; ACB, Anticholinergic Cognitive Burden; ASA, American Society of Anesthesiologists; BMI, body mass index | | | | | | |
